# Reduced Oligodendrocyte Density and Axonal Caliber Associated With Mitochondrial Alterations in the White Matter of Chronically‐Starved Mice

**DOI:** 10.1002/eat.70036

**Published:** 2026-01-16

**Authors:** Stephan Lang, Annelie Zimmermann, Kaja Dickert, Hanna Rupprecht, Julia Priebe, Fabienne Haberland, Hanna‐Sophia Henschke, Katharina Schuster, Marcus Frank, Linda Frintrop

**Affiliations:** ^1^ Institute of Anatomy, Rostock University Medical Center Rostock Germany; ^2^ Medical Biology and Electron Microscopy Center, Rostock University Medical Center Rostock Germany; ^3^ Department Life, Light and Matter University of Rostock Rostock Germany

**Keywords:** anorexia nervosa, axons, chronic starvation, glial cells, mitochondria, myelinated nerve fibers, oligodendrocytes, starvation‐induced hyperactivity, white matter

## Abstract

**Objective:**

Anorexia nervosa (AN) is a severe eating disorder associated with extreme weight loss, hyperactivity, and amenorrhea. Neuroimaging studies revealed brain atrophy and disruption of white matter integrity in the corpus callosum (CC) of patients with AN. However, the underlying pathophysiological mechanisms remain unclear. Emerging evidence indicates that starvation induces changes in mitochondrial metabolism and dynamics. We hypothesize that disturbances in white matter integrity arise from modifications in oligodendrocytes, associated with changes in the morphology of myelinated fibers and mitochondrial structure.

**Method:**

The starvation‐induced hyperactivity (SIH) model was used, in which mice received a restricted daily amount of food in combination with free access to a running wheel. A body weight loss of 25% was maintained over 2 weeks, followed by a 3‐week refeeding phase. Oligodendrocyte density and staining intensity of oligodendrocyte lineage transcription factor 2 (OLIG2) in the CC were analyzed using immunohistochemical staining. Morphometric investigation of myelinated fibers and mitochondria was conducted by transmission electron microscopy (TEM) analysis.

**Results:**

Starvation led to decreased oligodendrocyte density and reduced anti‐OLIG2 staining intensity in the CC, which was reversible following refeeding. Additionally, starvation induced a decrease in axonal caliber and an increase in mitochondrial density in the white matter, accompanied by a reduction of mitochondrial area.

**Discussion:**

The findings suggest that oligodendroglial and axonal alterations, alongside disrupted mitochondrial dynamics, impair structural integrity in the white matter and contribute to the pathophysiology of AN.

## Summary

- Anorexia nervosa (AN) is associated with disrupted white matter integrity as well as alterations of mitochondrial metabolism. However, the underlying pathophysiological mechanisms remain poorly understood.
- This study utilized a murine starvation model mimicking AN to investigate the consequences of starvation on oligodendrocytes, myelinated fibers, and mitochondria in the brain.
- The findings revealed that starvation induces a decrease in oligodendrocyte density as well as axonal caliber and an increase in mitochondrial density, accompanied by a reduction in mitochondrial area, indicating that white matter and mitochondria dysfunction play a role in the pathology of AN.

## 1 Introduction

Anorexia nervosa (AN) is a severe psychiatric and eating disorder characterized by body weight loss, hyperactivity, and amenorrhea (Herpertz‐Dahlmann 2015; Moskowitz and Weiselberg 2017). The lifetime prevalence of AN is approximately 4%, with adolescent females being predominantly affected (Silén and Keski‐Rahkonen 2022; van Eeden et al. 2021). Due to limited treatment options, patients with AN exhibited poor remission rates of 52%, coupled with a relapse rate of 26%, and one of the highest mortality rates among all psychiatric disorders (Chesney et al. 2014; Miskovic‐Wheatley et al. 2023). Furthermore, magnetic resonance imaging studies demonstrated reductions in the brain volume of gray and white matter, which is associated with cognitive impairments, such as deficits in logical reasoning (Castro‐Fornieles et al. 2010; McCormick et al. 2008; Seitz et al. 2018; Walton et al. 2022). Additionally, diffusion tensor imaging studies have indicated disrupted integrity, particularly in the corpus callosum (CC), suggesting altered axonal and myelin sheaths morphology (Barona et al. 2019; Griffiths et al. 2021; Laczkovics et al. 2022; Miles et al. 2020; Nickel et al. 2019; von Schwanenflug et al. 2019; Travis et al. 2015; Zhang et al. 2020). Consistent with these findings, decreased fiber bundle volumes in the CC and hints for reduced myelin content in patients with AN have been observed (de La Cruz et al. 2023; Maier et al. 2022; Murray et al. 2023; Pappaianni et al. 2022; Travis et al. 2015).

Only a few studies have investigated post‐mortem brain tissue in patients with AN, revealing neuronal degeneration and altered dendritic morphology as well as astroglial density within the gray matter (Gaiaschi et al. 2024; Kawakami et al. 2022; Martin 1958; Neumärker et al. 1997). Furthermore, reduced serum insulin‐like growth factor 1 (IGF‐1) levels during the acute phase of AN are well‐documented, suggesting impaired glial differentiation (Hsieh et al. 2004; Janowska et al. 2020; Keeler et al. 2022; Misra and Klibanski 2016). Despite the established findings of brain atrophy in AN, the underlying pathophysiological mechanisms and their potential long‐term functional consequences remain mostly unclear.

To investigate these mechanisms, we established the starvation‐induced hyperactivity (SIH) model in mice (Staffeld et al. 2023). In this model, each animal received an individually calculated, restricted amount of food per day over a time span of 3 weeks, resulting in a 25% body weight loss while having free access to a running wheel (Frintrop, Liesbrock, et al. 2018; Staffeld et al. 2023). This allows for mimicking the predominantly chronic course of AN (Miskovic‐Wheatley et al. 2023). We have demonstrated that chronic starvation leads to decreased CC volume and oligodendrocyte density in the CC, confirmed by another study using a different AN animal model (Frintrop, Trinh, et al. 2018; Verspohl et al. 2025; Zimmermann et al. 2023, 2025). Moreover, chronic starvation has been shown to increase serum neurofilament light chain (NfL) levels, a marker of neuronal damage (Zimmermann et al. 2025). To the best of our knowledge, no investigations on the integrity of myelinated fibers in AN animal models exist to date. However, older studies, primarily conducted in the developing rat brain, reported impaired myelin synthesis and reduced axonal caliber as a consequence of undernutrition (Delaney et al. 1981; Faúndez et al. 1990; Fishman et al. 1971; Reddy et al. 1979; Royland et al. 1992; Wiggins et al. 1974; Yusuf et al. 1981).

Emerging evidence suggested that oxidative stress plays a major role in the pathophysiology of AN, as several studies have demonstrated increased oxidative stress markers alongside reduced antioxidant capacities in affected patients (Amerio et al. 2024; Gaiaschi et al. 2024; Kovalčíková et al. 2021; Solmi et al. 2016). Mitochondria represent the primary source of reactive oxygen species (ROS) (reviewed in Balaban et al. 2005). Investigations conducted in post‐mortem and AN animal studies indicated altered mitochondrial metabolism and dynamics (fission and fusion), which could contribute to enhanced ROS production (Bhasin et al. 2023; Gaiaschi et al. 2024; Hurley et al. 2021; Nobis et al. 2018; Spero et al. 2024). Mitochondrial fission and fusion are tightly regulated processes that enable mitochondria to adapt to cellular metabolic demands and support key cellular functions. Fission divides one mitochondrion into two, facilitating the removal of damaged mitochondria, whereas fusion preserves mitochondrial functionality by combining mitochondrial contents (reviewed in Chen et al. 2023; Tábara et al. 2025). Mitochondrial fission can be triggered by oxidative stress, but can also contribute to the production of ROS (reviewed in Ježek et al. 2018). Moreover, fission promotes apoptosis and is therefore considered a marker for mitochondrial stress (Frank et al. 2001). Notably, increased mitochondrial fission analyzed by protein expression has been observed in AN animal models (Bhasin et al. 2023; Hurley et al. 2021; Nobis et al. 2018). To the best of our knowledge, no electron microscopical analyses of axons and mitochondria in the white matter have been performed in AN animal models so far.

Therefore, we hypothesized that disturbances in white matter integrity arise from a reduction in oligodendrocyte density, accompanied by changes in the morphology of myelinated fibers and mitochondria. This study investigated whether chronic starvation and subsequent refeeding alter oligodendrocyte density within the CC. Furthermore, we present the first ultrastructural analyses of myelinated fibers and mitochondrial morphology within white matter using the SIH model. Understanding these cellular alterations may provide insights into cognitive and neurological deficits observed in patients with AN.

## 2 Materials and Methods

### 2.1 Animals

Female 4‐week‐old C57BL/6J mice (*n* = 81) were obtained from Janvier Labs (Le Genest‐Saint‐Isle, France). The mice were housed in individual cages with unrestricted access to a running wheel for the entire duration of the experiment under a light/dark cycle of 12/12 h (lights on at 6 AM) and at a temperature of 22°C ± 2°C. Cages were changed weekly with fresh bedding and a water bottle. Furthermore, microbiological monitoring was conducted based on the Federation of European Laboratory Animal Science Associations (FELASA) recommendations. The animal studies were approved by the Review Boards for the Care of Animal Subjects of the district government of Mecklenburg‐Western Pomerania (reference number 7221.3‐1‐005/21).

### 2.2 Study Design

**FIGURE 1:**
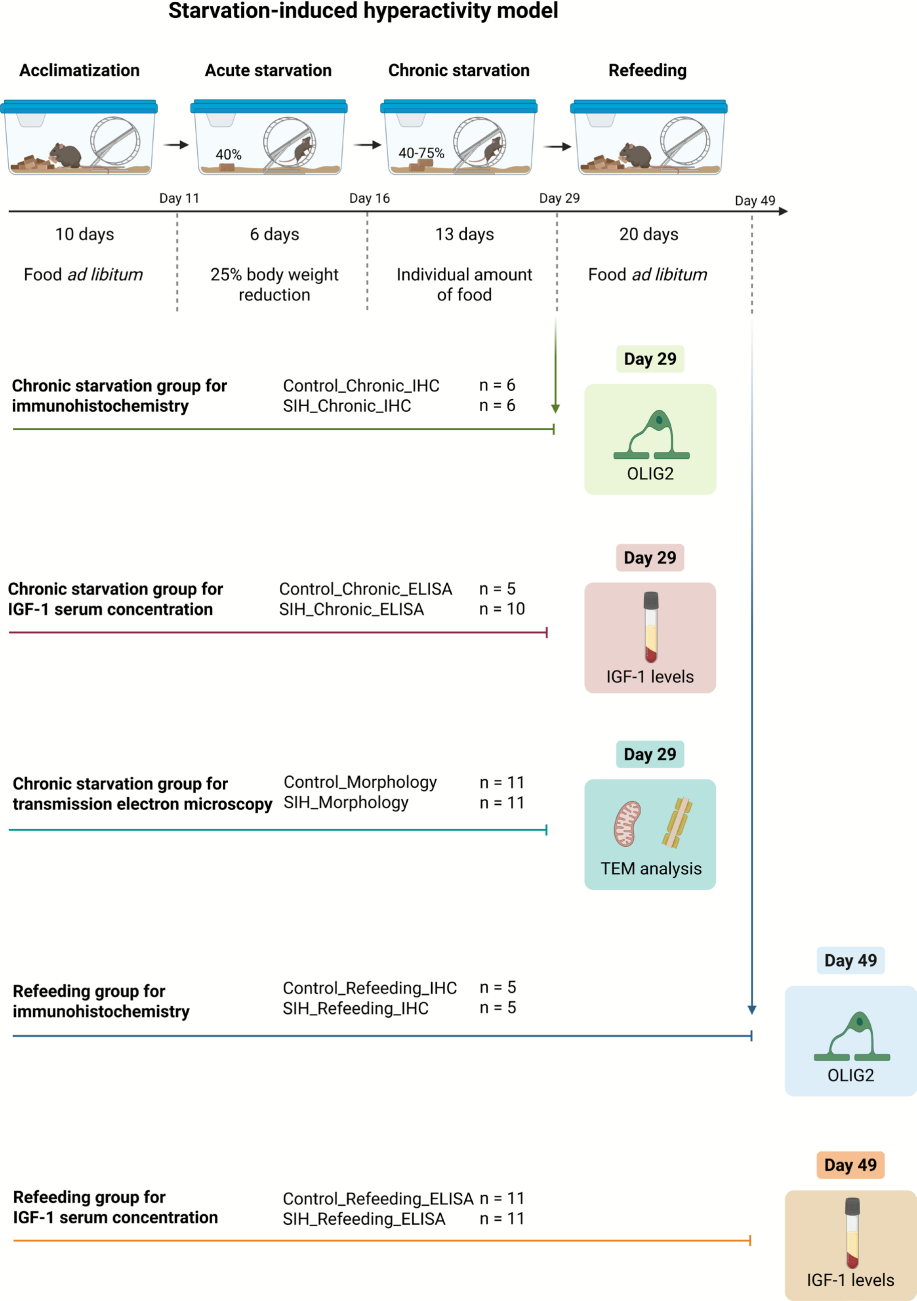
Schematic illustration of the experiment set‐up, including cohorts with total sample sizes and the corresponding methods in the present study. IGF‐1: Insulin‐like growth factor 1; OLIG2: Oligodendrocyte lineage transcription factor 2; SIH: Starvation‐induced hyperactivity; TEM: Transmission electron microscopy. Created with Biorender.com.

The SIH model was used as previously described by Frintrop et al. and Staffeld et al. (Frintrop, Liesbrock, et al. 2018; Staffeld et al. 2023). Initially, the mice were housed with *ad libitum* access to food (Ssniff, Soest, Germany) and water during a 10‐day acclimatization phase. Body weight, food intake, and estrous cycle (via vaginal smears) were monitored daily at 1 PM. Following the acclimatization phase, mice were randomly assigned to either the SIH or control group (Control_Chronic_IHC: *n* = 6; SIH_Chronic_IHC: *n* = 6; Control_Chronic_ELISA: *n* = 5; SIH_Chronic_ELISA: *n* = 10; Control_Morphology: *n* = 11; SIH_Morphology: *n* = 11; Control_Refeeding_IHC: *n* = 5; SIH_Refeeding_IHC: *n* = 5; Control_Refeeding_ELISA: *n* = 11; SIH_Refeeding_ELISA: *n* = 11; Figure 1). Some mice had to be finalized and excluded prior to the experiment's end point due to exhibiting termination criteria (SIH_Chronic_ELISA: *n* = 1; SIH_Morphology: *n* = 1; SIH_Refeeding_ELISA: *n* = 1). During the acute starvation phase, SIH mice received 40% of their baseline food intake (defined as the average food intake during the acclimatization phase) until a 25% body weight loss was achieved. The acute starvation phase was defined as a 1‐week phase. After reaching the target weight, food intake was adjusted to 45%–70% of their baseline food intake to maintain the 25% body weight loss for an additional 2 weeks, thereby mimicking chronic starvation. Food for SIH mice was provided daily at 1 PM. During the subsequent 3‐week period of the refeeding phase, SIH mice obtained *ad libitum* food. Control mice had *ad libitum* access to food throughout the whole experiment. Estrous cycle and running wheel activity were monitored as previously reported (further details: Data S1) (Frintrop, Liesbrock, et al. 2018; Gabloffsky et al. 2022; Staffeld et al. 2023). Termination criteria included a 10% additional weight loss within 24 h, presence of cramps, paralysis, breathing noises, or forced breathing.

### 2.3 Immunohistochemistry and Image Analysis

For immunohistochemistry analysis, the mice were euthanized via intraperitoneal injection of ketamine (100 mg/kg) and xylazine (10 mg/kg) at the end of the experiment (Control_Chronic_IHC: *n* = 6; SIH_Chronic_IHC: *n* = 6; Control_Refeeding_IHC: *n* = 5; SIH_Refeeding_IHC: *n* = 5). Transcardial perfusion was performed using phosphate‐buffered saline, followed by tissue fixation using 3.7% paraformaldehyde solution (pH 7.4). Brains were paraffin‐embedded and sectioned coronally at 5 μm‐thickness. Sections were processed following established standard protocols (Beecken et al. 2023) (Table 1, further details: Data S1). Investigations were performed in Region 215 according to the Mouse Brain Atlas from Sidman et al. (https://www.hms.harvard.edu/research/brain/atlas.html), corresponding to Bregma +0.14 in Paxinos and Franklin's Mouse Brain Atlas (Paxinos and Franklin 2001).

**TABLE 1:** Antibodies used for immunohistochemistry.

Antigen	Species	Dilution	Clonality	Purchase number	RRID	Supplier
Primary antibodies						
OLIG2	Rabbit	1:1000	Polyclonal	AB9610	AB_570666	Sigma Aldrich, Germany
Secondary antibodies						
Anti‐rabbit IgG	Goat	1:200	Polyclonal	BA‐1000	AB_2313606	Vector Laboratories, USA

Two brain sections per animal were digitized by using a Grundium Ocus 40 digital slide scanner (Grundium, Tampere, Finland). Cell quantification and staining intensity measurement were performed using the software QuPath Version 0.5.1 (Bankhead et al. 2017). For cell density measurements, oligodendrocyte lineage transcription factor 2‐positive (OLIG2^+^) cells were quantified in the whole CC of one brain hemisphere as region of interest. Cell counts in cells per mm^2^ were conducted by two evaluators blinded to the treatment groups and averaged per animal. Staining intensity measurement, assessed as OLIG2^+^ stained area within the region of interest and presented as relative cell area per total area, was performed by applying a pixel classifier and creating the best fitting threshold (further details: Data S1).

### 2.4 Measurement of Serum IGF‐1

At the end of the chronic starvation and refeeding phase, blood samples were collected retro‐orbital for the analysis of serum IGF‐1 (Control_Chronic_ELISA: *n* = 5; SIH_Chronic_ELISA: *n* = 8; Control_Refeeding_ELISA: *n* = 11; SIH_Refeeding_ELISA: *n* = 8). The collected blood samples were centrifuged at 3400*g* for 15 min at 4°C, and supernatant serum was obtained. Serum IGF‐1 concentrations were measured using the ELISA Luminex Discovery Assay (R&D‐Systems LXSAMSM, Minneapolis, MN, USA) according to manufacturer instructions via flow cytometry (Luminex100/200, Merck, Darmstadt, Germany). Some animals had to be excluded due to insufficient serum amounts (SIH_Chronic_ELISA: *n* = 1; SIH_Refeeding_ELISA: *n* = 2).

### 2.5 Ultrastructural Analysis

For transmission electron microscopy (TEM) analyses, three randomly selected mice each from the control and SIH group of the Morphology cohort were used (Control_Morphology: *n* = 3; SIH_Morphology: *n* = 3). These mice were euthanized via intraperitoneal injections of ketamine (100 mg/kg) and xylazine (10 mg/kg) following the chronic starvation phase. Transcardial perfusion was performed using Ringer's solution supplemented with sodium nitrite and heparin, followed by fixation with 2% glutaraldehyde and 1.4% paraformaldehyde solution (pH 7.4). After dissection, the brains were embedded in 2.5% agarose. Coronal brain sections of 300 μm thickness were prepared using a vibratome (Leica VT1000 S, Leica, Wetzlar, Germany). The sections were then processed for TEM analysis using standard protocols (further details: Data S1). The Cingulum (Cg) as a white matter structure adjacent to the CC was selected as a region of interest due to the predominance of perpendicular axonal cross‐sections in this area. Investigations were performed in the Cg of Region 265 according to the Mouse Brain Atlas from Sidman et al. (https://www.hms.harvard.edu/research/brain/atlas.html), corresponding to Bregma −0.94 in Paxinos and Franklin's Mouse Brain Atlas (Paxinos and Franklin 2001).

TEM Imaging of ultrathin sections was performed using a Zeiss EM902 (Carl Zeiss Microscopy, Jena, Deutschland) and a field emission scanning microscope (Zeiss Merlin VP compact, Carl Zeiss Oberkochen, Germany) (further details: Data S1). Three to four images per animal were evaluated and averaged. The morphometric analyses of myelinated fibers and mitochondria in the Cg were conducted blinded to the treatment groups using the software QuPath Version 0.5.1 (Bankhead et al. 2017). For myelinated fibers, axonal and fiber cross‐sectional areas were measured to calculate axonal diameter, myelinated fiber diameter, G‐Ratio (1), and myelin thickness (2).

(1) $$G-\text{Ratio}=\frac{\text{axonal area}}{\text{myelinated fiber area}}$$

(2) $$\text{Myelin thickness}=\frac{\text{myelinated fiber diameter}-\text{axonal diameter}}{2}$$

For morphometric analysis, a rectangular grid (8 × 8 μm) was superimposed across each image, and the analysis was conducted on every tenth grid frame. Additionally, the circularity (3) was assessed, and diagonally oriented axons with a circularity below 0.7 were excluded from the investigation. In total, 300 to 400 myelinated fibers per animal were measured.

(3) $$\text{Circularity}=\frac{4\pi \times \text{axonal area}}{{\text{axonal perimeter}}^{2}}$$

For mitochondrial evaluation, the mitochondrial density was quantified as number of mitochondria per mm^2^ tissue. Morphometric analysis of mitochondrial cross‐sectional area was performed, and the relative mitochondrial area (total mitochondrial area relative to total tissue area) was calculated on every fifth grid frame. The change in mitochondrial area is expected to lead to an altered mitochondrial density due to increased or decreased encounters of mitochondrial cross sections. Based on Disector's Principle, we estimated the expected mitochondrial density decrease based on the reduction in mitochondrial area and then used this as a correction factor to determine the increase in mitochondria density (Cruz‐Orive 1987) (further details: Data S1). Myelinated fibers were excluded from the evaluated area and mitochondria within the axons were measured separately. In total, 500 to 750 mitochondria per animal were morphometrically assessed.

### 2.6 Statistics

Data are presented as means ± standard error of the mean (SEM). First, the Grubbs' test (*α* = 0.05) was performed to detect outliers, followed by the Shapiro–Wilk test to check for normal distribution and the Levene Test for homogeneity of variance. For the statistical analysis of body weight and running wheel activity, the values measured in the distinct phases were compared: acclimatization phase (days 1–10), acute starvation phase (days 11–16), and chronic starvation phase (days 17–29). The evaluation of body weight and running wheel activity between SIH and control mice within each phase was performed using two‐way ANOVA with repeated measurements and a significance level *p* ≤ 0.05. For post hoc evaluations, Bonferroni correction was used. To investigate whether the estrous cycle differed between the control and SIH groups, the presence or absence of the fertile phase in each 4‐day block was assessed using the *χ* ^2^ test. For immunohistochemical staining, serum IGF‐1 concentration, and TEM evaluation, two‐sided Student's *t*‐test was used if the data were normally distributed; otherwise, the Mann–Whitney test was applied. The significance level was determined as *p* ≤ 0.05. Effect sizes were calculated as Cohen's d. Statistical analysis was performed with SPSS version 20 (IBM, Chicago, IL, USA) and GraphPad Prism 10.2 (GraphPad Software, Boston, MA, USA). The sample size was calculated using an a priori *one‐way* ANOVA with the G*Power Software 3.1.4 (Faul et al. 2007) (further details: Data S1).

## 3 Results

### 3.1 Chronic Starvation Leads to the AN‐Related Symptoms of Hyperactivity and Amenorrhea

**FIGURE 2:**
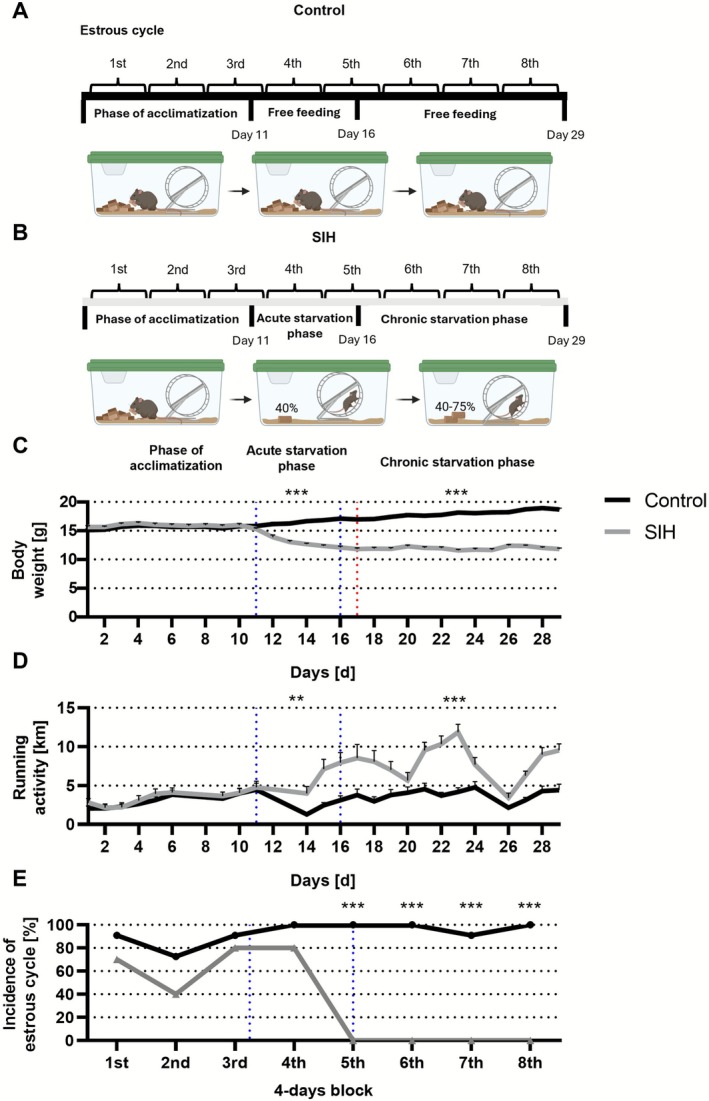
Chronic starvation induces AN‐related symptoms, such as hyperactivity and amenorrhea. (A, B) The experimental setup with the (A) control and (B) SIH group is schematically illustrated. Created with BioRender.com. (C–E) Measurement of body weight, running wheel activity and incidence of estrous cycle within 4‐days blocks was conducted daily at 1 PM. The blue dotted lines indicate the beginning of the acute and chronic starvation phase. The red dotted line highlights the time point when the SIH group reached a 25% body weight loss. (C, D) To investigate body weight loss and running wheel activity between control and SIH mice, two‐way ANOVA with repeated measurements was performed for each phase. (E) To compare the incidence of estrous cycle between control and SIH mice, the *χ* ^2^ test was conducted for each block separately. ** = *p* ≤ 0.01; *** = *p* ≤ 0.001.

First, we investigated whether chronic starvation induces AN‐related symptoms (hyperactivity and amenorrhea) in the cohort used for morphological analyses (Control_Morphology: *n* = 11; SIH_Morphology: *n* = 10, Figure 2). On average, SIH mice reached a 25% body weight loss by day 17. The body weight of SIH mice was reduced compared to control mice in the acute and chronic starvation phase (acute starvation phase: Control_Morphology: 16.47 g ± 0.24 vs. SIH_Morphology: 13.22 g ± 0.22; *p* = < 0.001; Cohen's d: −3.992; chronic starvation phase: Control_Morphology: 17.96 g ± 0.23 vs. SIH_Morphology: 11.95 g ± 0.17; *p* = < 0.001; Cohen's d: −8.184; Figure 2C). Moreover, acute and chronic starvation induced hyperactivity in SIH mice (acute starvation phase: Control_Morphology: 2.83 km ± 0.45 vs. SIH_Morphology: 5.95 km ± 0.85; *p* = 0.004; Cohen's d: 1.203; chronic starvation phase: Control_Morphology: 3.81 km ± 0.46 vs. SIH_Morphology: 8.07 km ± 0.52; *p* = < 0.001; Cohen's d: 1.601; Figure 2D). Throughout the complete chronic starvation phase, a fertile estrous phase was absent in SIH mice, indicating amenorrhea (*χ* ^2^ test, exemplary for the 5th and 8th block: Control_Morphology vs. SIH_Morphology: *χ*(df) = 1; *χ* ^2^ = 21; *p* = < 0.001; Figure 2E). In summary, chronic starvation led to the AN‐related symptoms of hyperactivity and amenorrhea. The other mouse cohorts included in this study were previously used in our investigations; Body weight, running wheel activity and estrous cycle data have been published in (Staffeld et al. 2023; Zimmermann et al. 2025) (further details: Data S1).

### 3.2 Chronic Starvation Induces a Reduction in the Density of OLIG2 ^+^ Cells as Well as OLIG2 Staining Intensity in the CC and Serum IGF‐1

**FIGURE 3:**
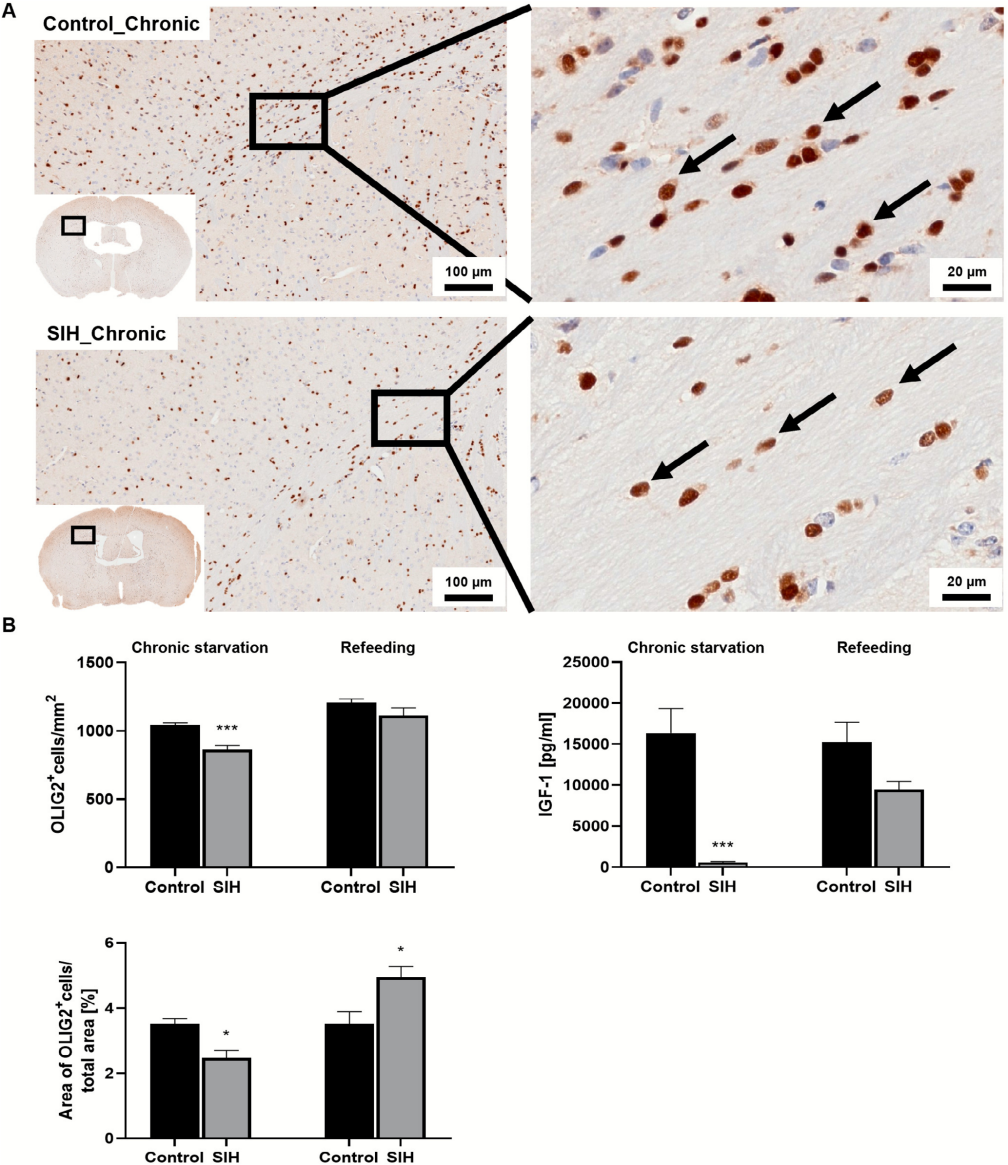
Chronic starvation induces decreased oligodendrocyte density in the CC, which normalizes after refeeding. (A) OLIG2^+^ cell densities in an exemplary low magnification (left) and high magnification (right) IHC image of the CC. OLIG2^+^ cells are indicated by black arrows. (B) After chronic starvation, a decrease in oligodendrocyte density, OLIG2 staining intensity and serum IGF‐1 concentration was detected. Oligodendrocyte density, OLIG2 staining intensity and serum IGF‐1 concentration normalized after refeeding. Two‐sided Student's *t*‐test, * = *p* ≤ 0.05; *** = *p* ≤ 0.001.

Further, we evaluated whether chronic starvation induces a change in oligodendrocyte density and OLIG2 staining intensity in the CC, and whether these effects were reversible following refeeding (Figure 3). After chronic starvation, SIH mice exhibited a reduction in oligodendrocyte density compared to control mice (Control_Chronic_IHC: 1041 cells/mm^2^ ± 18.64 vs. SIH_Chronic_IHC: 864 cells/mm^2^ ± 27.02; *p* = < 0.001; Cohen's d: −3.163). This observation was accompanied by a decrease in OLIG2 staining intensity (Control_Chronic_IHC: 3.51% ± 0.16 vs. SIH_Chronic_IHC: 2.47% ± 0.22; *p* = 0.015; Cohen's d: −2.223). However, oligodendrocyte density did not differ between the two groups after refeeding (Control_Refeeding_IHC: 1208 cells/mm^2^ ± 25.51 vs. SIH_Refeeding_IHC: 1114 cells/mm^2^ ± 54.41; *p* = 0.155; Cohen's d: −0.989). The OLIG2 staining intensity in SIH mice was increased after refeeding compared to controls (Control_Refeeding_IHC: 3.51% ± 0.37 vs. SIH_Refeeding_IHC: 4.95% ± 0.32; *p* = 0.0198; Cohen's d: 1.83). Next, we investigated whether the observed changes in oligodendrocyte density could be due to an altered serum IGF‐1 concentration as an essential growth factor for the differentiation of glial cells and axon development (Guo et al. 2018; Hsieh et al. 2004; Janowska et al. 2020) (Figure 3). Chronic starvation led to a reduction in serum IGF‐1 concentration in SIH mice (Control_Chronic_ELISA: 16336 pg/mL ± 3003 vs. SIH_Chronic_ELISA: 526.4 pg/mL ± 125; *p* = 0.002; Cohen's d: −3.895). After refeeding, no difference in IGF‐1 concentration between SIH and control mice was observed (Control_Refeeding_ELISA: 15235 pg/mL ± 2444 vs. SIH_Refeeding_ELISA: 9496 pg/mL ± 970; *p* = 0.073; Cohen's d: −0.888). In summary, chronic starvation induced a decrease in oligodendrocyte density and OLIG2 staining intensity in the CC as well as serum IGF‐1 concentration, whereas these parameters normalized following refeeding.

### 3.3 Chronic Starvation Induces a Reduction in Axon Caliber of Myelinated Fibers in the Cg

**FIGURE 4:**
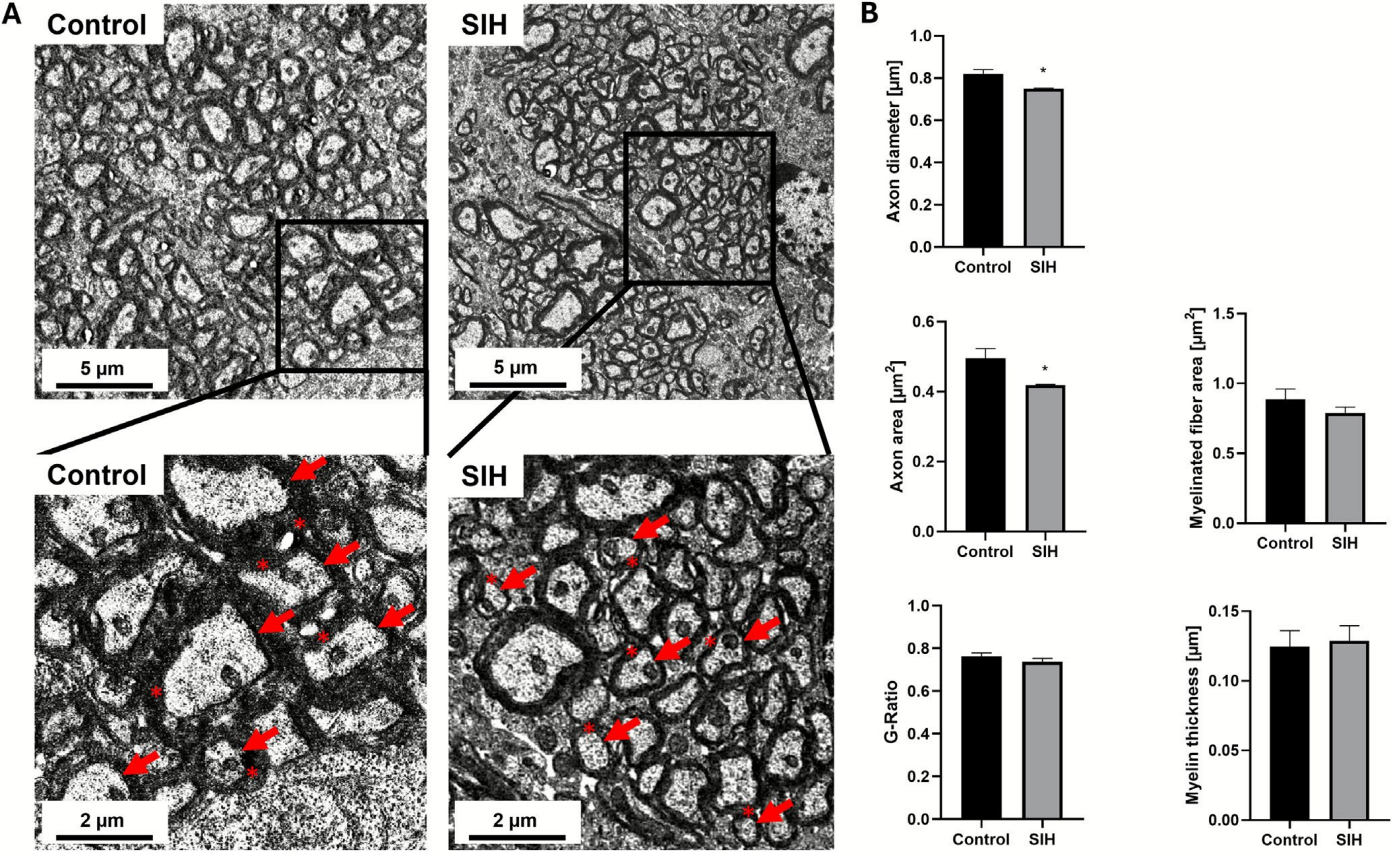
Chronic starvation induces a decrease in axon diameter and area in the Cg. (A) In the exemplary low magnification TEM images (above) and high magnification images (below), myelinated fibers are displayed. The red arrows indicate axons and the asterisks the myelin sheath. (B) A decrease in axon area and diameter in the SIH mice was detected after starvation, whereas the myelin fiber area, the G‐Ratio, and myelin thickness in the Cg were not affected. Two‐sided Student's *t*‐test was conducted for all analyses, except the myelin fiber area, which was evaluated using the Mann–Whitney test. * = *p* ≤ 0.05.

Next, we investigated whether chronic starvation affects axon and myelin morphology of myelinated fibers in TEM images of the Cg, as an additional white matter structure (Figure 4). Starvation induced a decrease in axonal diameter (Control_Morphology: 0.821 μm ± 0.019 vs. SIH_Morphology: 0.75 μm ± 0.003; *p* = 0.022; Cohen's d: −3.008) and axonal area (Control_Morphology: 0.496 μm^2^ ± 0.027 vs. SIH_Morphology: 0.419 μm^2^ ± 0.002; *p* = 0.046; Cohen's d: −2.362). In contrast, the area of the myelinated fibers did not differ between groups after starvation (Control_Morphology: 0.886 μm ± 0.074 vs. SIH_Morphology: 0.789 μm ± 0.041; *p* = 0.4; Cohen's d: −0.929). Furthermore, neither the G‐Ratio (Control_Morphology: 0.763 ± 0.016 vs. SIH_Morphology: 0.737 ± 0.016; *p* = 0.311; Cohen's d: −0.963) nor the myelin thickness (Control_Morphology: 0.125 μm ± 0.012 vs. SIH_Morphology: 0.129 μm ± 0.01; *p* = 0.807; Cohen's d: 0.205) were altered between SIH and control mice. In summary, chronic starvation induced a decrease in axon caliber, while the G‐Ratio and myelin thickness remained unaffected.

### 3.4 Chronic Starvation Induces an Increase in Mitochondrial Density Paralleled by Decreased Mitochondrial Area in the Cg Indicating Enhanced Mitochondrial Fission

**FIGURE 5:**
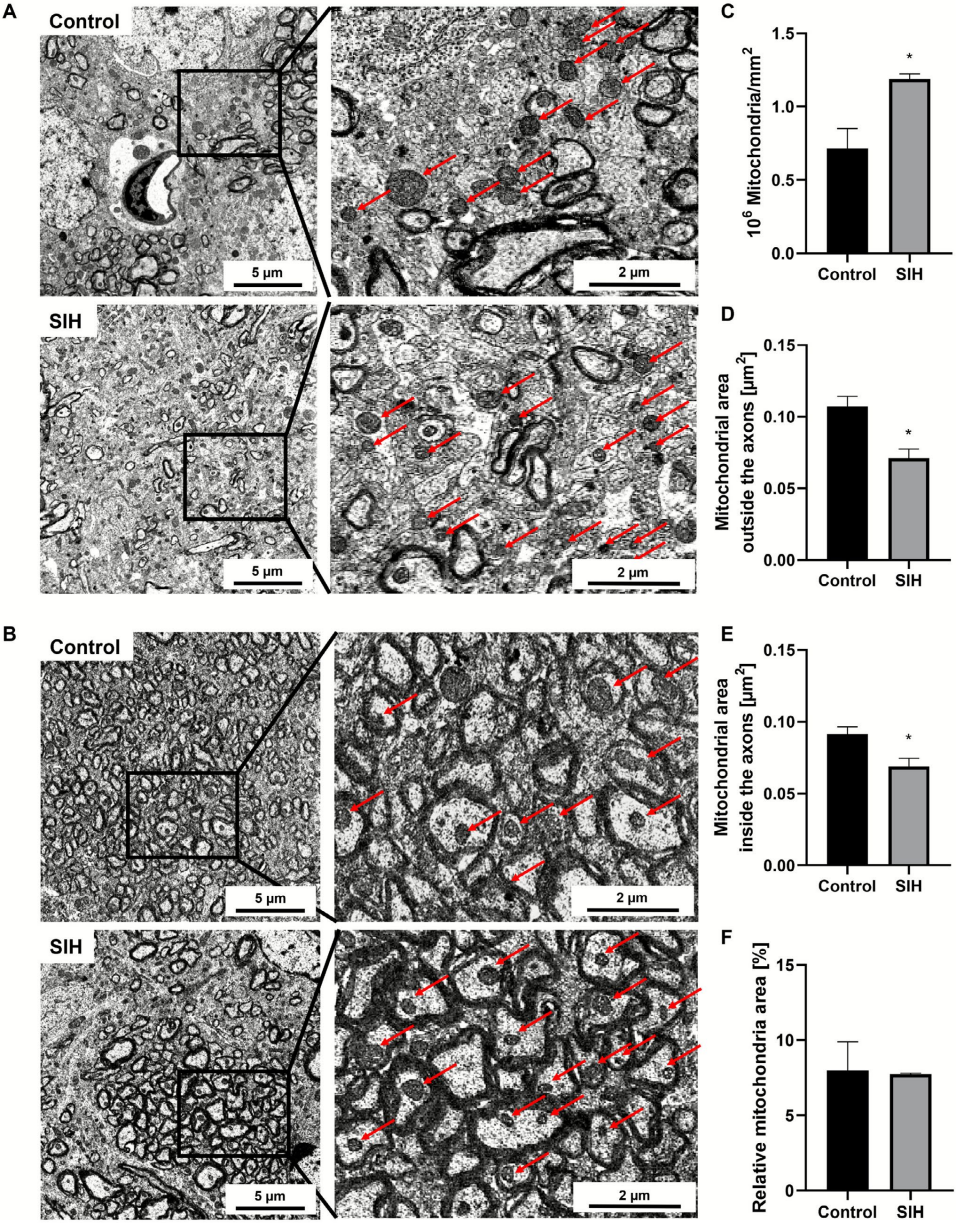
Chronic starvation induces morphological alterations in mitochondria of the Cg. (A, B) In the exemplary low magnification TEM images (left) and high magnification TEM images (right), mitochondria (A) outside and (B) inside the axons in the Cg are displayed. Red arrows indicate mitochondria. (C–F) After starvation, the mitochondrial density in SIH mice increased, paralleled by a reduction in mitochondrial area outside and inside the axons in the Cg. Two‐sided Student's *t*‐test, * = *p* ≤ 0.05.

We investigated whether chronic starvation induces subcellular morphological alterations of mitochondria in the Cg, assessed via TEM imaging (Figure 5). Chronic starvation led to an increase in mitochondria density in SIH mice compared to control (Control_Morphology: 0.715 × 10^6^ mitochondria/mm^2^ ± 0.136 × 10^6^ vs. SIH_Morphology: 1.188 × 10^6^ mitochondria/mm^2^ ± 0.036 × 10^6^; *p* = 0.028; Cohens' d: 2.739). Using the Disector's Principle, the expected mitochondrial density based on the altered area was taken into account, and an increase of 150% in mitochondrial density in SIH mice was estimated (expected mitochondrial density: 0.474 × 10^6^ mitochondria/mm^2^). This was paralleled by a decrease in mitochondrial area outside the axons (Control_Morphology: 0.107 μm ± 0.007 vs. SIH_Morphology: 0.071 μm ± 0.006; *p* = 0.019; Cohen's d: −3.127) as well as inside the axons (Control_Morphology: 0.091 μm ± 0.005 vs. SIH_Morphology: 0.069 μm ± 0.006; *p* = 0.043; Cohen's d: −2.313). No change in relative mitochondria area in SIH mice could be observed after chronic starvation (Control_Morphology: 8% ± 1.89 vs. SIH_Morphology: 7.749% ± 0.057; *p* = 0.903; Cohen's d: −0.108). In summary, chronic starvation induced an increase in mitochondrial density accompanied by a decrease in mitochondrial area, potentially indicating enhanced mitochondrial fission events.

## 4 Discussion

The pathophysiology underlying the brain atrophy in AN remains poorly understood. To elucidate potential mechanisms in the CC, we demonstrated that chronic starvation with 25% weight loss induced a reduction of oligodendrocyte density and anti‐OLIG2 staining intensity. This finding is consistent with previous studies (Verspohl et al. 2025; Zimmermann et al. 2023). OLIG2 is continuously expressed throughout the oligodendrocyte lineage, including oligodendrocyte precursor cells (OPCs), premyelinating oligodendrocytes (preOLs), and the differentiated myelinating oligodendrocytes (OLs) (reviewed in Baumann and Pham‐Dinh 2001). In previous studies, oligodendrocyte density assessed via adenomatous polyposis coli antigen, a specific marker for OLs, remained unaltered after chronic starvation (Frintrop et al. 2019; Verspohl et al. 2025). This suggests that primarily OPCs and preOLs, rather than mature OLs, are affected by chronic starvation. One of our previous studies and others demonstrated reduced cell proliferation after starvation, while apoptosis in the CC remained unaffected (Barbarich‐Marsteller et al. 2013; Frintrop et al. 2019; Verspohl et al. 2025). Furthermore, the OL population exhibits a long lifespan under physiological conditions, particularly in the CC (Tripathi et al. 2017). Therefore, impaired proliferation and differentiation of OPCs are likely to contribute to the observed reduction of OLIG2^+^ cell density after chronic starvation.

Reduced OLIG2 staining intensity after starvation indicates changed OLIG2 expression and/or morphology of oligodendrocytes. Following refeeding, oligodendrocyte density was normalized, while OLIG2 staining intensity even increased in SIH mice, indicating that the observed alterations after chronic starvation were reversible. Since OLIG2 is a key transcription factor regulating oligodendrocyte development and differentiation, its expression may be upregulated during refeeding as part of a recovery mechanism to restore oligodendrocyte populations (Mei et al. 2013). Alternatively, the increased OLIG2 staining intensity could reflect morphological alterations of oligodendrocytes during recovery.

Next, decreased serum IGF‐1 levels following chronic starvation were observed, which normalized after refeeding. This finding is in line with investigations in patients with AN and AN animal models (Misra and Klibanski 2016; Zgheib et al. 2014). IGF‐1 plays a crucial role in glial cell homeostasis by promoting the differentiation of progenitor cells into oligodendrocytes, supporting their maturation through enhancement of process branching and inhibiting apoptosis of OLs (Hsieh et al. 2004; Janowska et al. 2020; Mason et al. 2000). Further, IGF‐1 facilitates axon growth (Guo et al. 2018). The implications of reduced serum IGF‐1 concentration in the pathophysiology of AN remain mostly unexplored. Given the critical role of IGF‐1 in glial cell homeostasis, decreased IGF‐1 could contribute to the reduction in oligodendrocyte density and axon caliber.

Chronic starvation resulted in a decrease in axonal area and diameter, whereas no changes in myelinated fiber area, G‐Ratio and myelin thickness were detected in the Cg. The observed decrease in axonal caliber is in line with previous studies in patients with AN, which demonstrated reduced fiber bundle volume, respectively cross‐sectional area in the CC and Cg (Delaney et al. 1981; Faúndez et al. 1990; de La Cruz et al. 2023; Maier et al. 2022). Additionally, increased axonal diffusivity – a marker reflecting water molecule diffusion parallel to myelinated fibers– was observed in patients with AN. This increase was hypothesized to result from a reduction in axonal caliber, as smaller axons follow a more linear path with less tortuosity (Griffiths et al. 2021; de La Cruz et al. 2023; Miles et al. 2020). The decrease in axonal caliber detected in our study could support this hypothesis, since a decrease in axonal caliber is negatively correlated with the axonal diffusivity (Barazany et al. 2009; Takahashi et al. 2000). Moreover, elevated serum NfL concentrations in chronically‐starved mice, which, in combination with a reduced axon caliber, indicate axonal damage and atrophy (Reyes‐Ortega et al. 2020; Zimmermann et al. 2025). Notably, NfL is crucial for the growth of the axonal diameter (Sainio et al. 2021). Therefore, the decrease in axonal caliber represents a potential reason for the decrease in CC volume in SIH mice and white matter volume loss reported in patients with AN (Seitz et al. 2018; Zimmermann et al. 2025).

The unchanged myelin parameters contrast with studies in undernourished rats from birth over extended periods, which reported reduced myelin but did not utilize AN models (Delaney et al. 1981; Fishman et al. 1971; Reddy et al. 1979; Royland et al. 1992; Wiggins et al. 1974; Yusuf et al. 1981). Several neuroimaging studies in patients with AN revealed reduced white matter integrity and suggest decreased myelination (Griffiths et al. 2021; Laczkovics et al. 2022; Miles et al. 2020; Murray et al. 2023; Nickel et al. 2019; Pappaianni et al. 2022; von Schwanenflug et al. 2019; Travis et al. 2015). Since we found no evidence for myelin degradation in SIH mice, potentially a longer starvation period induces alterations in myelination. Myelin proteins, especially proteolipid protein and myelin basic protein, have long lives of several months (Lüders et al. 2019; Meschkat et al. 2022). Thus, rapid degeneration of myelin sheaths would likely be required to detect short‐term alterations caused by starvation. Consequently, starvation may primarily impair myelin synthesis leading to long‐term alterations but does not actively damage the myelin sheaths. Additionally, our study did not assess microstructural alterations such as the myelin lamellae or the rate of myelin synthesis.

This study provides the first morphological evidence of increased mitochondrial fission in an AN animal model, reflected by elevated mitochondrial density and reduced area outside and inside of axons. This finding is consistent with previous studies reporting increased levels of phosphorylated dynamin‐related protein 1 as a mitochondrial fission marker (Bhasin et al. 2023; Hurley et al. 2021; Nobis et al. 2018). These alterations suggest impaired energy metabolism in axons following starvation. Since mitochondria are crucial for axonal development and maintenance, mitochondrial dysfunction may contribute to the observed decrease in axonal caliber. This is supported by findings linking increased mitochondrial fission with axonal injury (reviewed in Pozo Devoto et al. 2022). Additionally, myelination is a high‐energy‐demanding process, which heavily depends on intact mitochondrial function, indicating possibly impaired myelination of axons due to starvation conditions (reviewed in Meyer et al. 2017). The observed mitochondrial phenotype may indicate mitochondrial stress. Zheng et al. demonstrated that hypoglycemia is a potent inducer of mitochondrial fission in fission yeast, linked to ROS production in fragmented mitochondria, and concluded the increase in ROS to be potentially due to mitochondrial fragmentation (Zheng et al. 2019). Notably, hypoglycemia is frequently observed in patients with AN and was reported in one of our previous studies (Gibson et al. 2020; Staffeld et al. 2023; Uotani et al. 2022). Thus, hypoglycemia represents a potential mechanism contributing to the detected increase in mitochondrial fission and may also be linked with changes in oligodendrocytes. Since especially OPCs depend mostly on mitochondrial oxidative phosphorylation, they are susceptible to hypoglycemia, which has been demonstrated to impair their proliferation, maturation, and survival (Rinholm et al. 2011; Rone et al. 2016; Yan and Rivkees 2006). Moreover, mitochondria may play a crucial role in oligodendrocyte differentiation as impaired oxidative phosphorylation inhibited oligodendroglial differentiation in vitro (reviewed in Gil and Gama 2023; see also Schoenfeld et al. 2010). If hypoglycemia is a key driver of mitochondrial alterations, associated oligodendroglial changes may be reversible upon refeeding.

Patients with AN exhibited decreased white matter volume, which was reversible upon weight rehabilitation (Seitz et al. 2018). Moreover, in patients, brain regions that are associated with higher oligodendrocyte specific gene expression showed a greater volume loss under starvation compared to regions with fewer oligodendrocyte specific gene expression, supporting a potential role of oligodendrocytes in brain volume reduction in AN (Bahnsen et al. 2022). This connection might link the normalization of oligodendrocyte density to an increase in white matter volume and normalization of microstructural alterations in patients with AN following weight rehabilitation (Griffiths et al. 2021; Maier et al. 2022; Nickel et al. 2019; von Schwanenflug et al. 2019; Seitz et al. 2018). We previously demonstrated that chronic starvation in mice led to a decrease in CC volume, which persisted after refeeding (Zimmermann et al. 2025). A longer refeeding period in mice may therefore be required to induce a normalization of white matter volume loss. The CC is thought to contribute to body image perception, which often is disturbed in patients with AN (Feusner et al. 2024; Gaudio and Quattrocchi 2012; Zhang et al. 2020). The Cg, a major white matter tract of the limbic system, connects the frontal, parietal and temporal lobes as well as the cingulate gyrus, thalamic nuclei, hippocampus, insula and amygdala (reviewed in Kollenburg et al. 2025). Moreover, the Cg has been shown to exhibit microstructural alterations and associated functional disturbances, including emotional regulation, memory, and reward processing in patients with AN (Bubb et al. 2018; Harrison et al. 2010; J. Keeler et al. 2021; Rye et al. 2025; Zhang et al. 2020). Thus, dysfunctions of oligodendrocytes and axons within the CC and Cg may underlie some psychopathological characteristics in AN. Another behavioral phenotype in patients with AN is excessive locomotor activity, which was also induced in SIH mice in this study (Kron et al. 1978; Mond and Gorrell 2021). Recent studies in ABA animals suggest that GABAergic synaptic plasticity in the hippocampus influences locomotor activity, with excessive activity enhancing α4βδ‐GABA_A_ receptor trafficking (reviewed in Aoki and Santiago 2022; Dong et al. 2025; see also Aoki et al. 2018).

It should be noted that in mice, the period between 3 weeks and 3 months of age represents a critical phase for brain maturation, during which the most development of myelinated fibers and oligodendrogliogenesis occurs (Balraj et al. 2022; Hammelrath et al. 2016; Psachoulia et al. 2009; Tripathi et al. 2017; Young et al. 2013). As starvation in SIH mice was induced at approximately 5 weeks of age, it likely interfered with these ongoing maturation processes, potentially affecting axonal and glial cell development. Similarly, early adolescence in humans constitutes a critical period for axonal growth and myelination, which may contribute to the detected white matter volume loss in patients with AN (Z. Chen et al. 2016; Genc et al. 2023; Lebel and Deoni 2018; Seitz et al. 2018; Yeung et al. 2014).

In summary, chronic starvation caused a reduction in oligodendrocyte density and anti‐OLIG2 staining intensity in the CC, alongside decreased serum IGF‐1 concentration, which normalized after refeeding. Additionally, chronic starvation induced a reduction in axonal area and diameter in the Cg. Further, chronic starvation led to an increase in mitochondrial density paralleled by reduced mitochondrial area. These alterations may contribute to brain atrophy and disturbance of white matter integrity in patients with AN. However, the underlying molecular mechanisms and potential functional implications require further evaluation. Moreover, TEM studies are required to analyze reversibility of ultrastructural alterations upon refeeding.

## Author Contributions

**Stephan Lang:** conceptualization, data curation, formal analysis, investigation, methodology, validation, visualization, writing – original draft, writing – review and editing. **Annelie Zimmermann:** data curation, formal analysis, investigation, methodology, validation, visualization. **Kaja Dickert:** data curation, formal analysis, investigation, methodology, visualization. **Hanna Rupprecht:** investigation. **Julia Priebe:** investigation. **Fabienne Haberland:** investigation. **Hanna‐Sophia Henschke:** investigation. **Katharina Schuster:** investigation. **Marcus Frank:** data curation, formal analysis, investigation, methodology, validation, visualization. **Linda Frintrop:** conceptualization, data curation, formal analysis, funding acquisition, investigation, methodology, project administration, resources, supervision, validation, visualization, writing – original draft, writing – review and editing. All authors have approved the final manuscript.

## Funding

This work was supported by the Doktor Robert Pfleger funding and intramural funding (FORUN program, University Medical Center Rostock).

## Ethics Statement

All animal procedures were conducted at the Institute of Anatomy at the Rostock University Medical Center, in accordance with EU Directive 2010/63 on the protection of animals used for scientific purposes and the recommendations of the Federation of European Laboratory Animal Science Associations (FELASA). The animal studies were approved by the Review Boards for the Care of Animal Subjects of the district government of Mecklenburg‐Western Pomerania (reference number 7221.3‐1‐005/21). The experiments were reported according to the ARRIVE (Animal Research: Reporting of In Vivo Experiments) guidelines (Du Percie Sert et al. 2020).

## Conflicts of Interest

The authors declare no conflicts of interest.

## Supporting information


**Data S1:** Supporting Information.

## Data Availability

The data that support the findings of this study are available from the corresponding author upon reasonable request.
