# Supplementary material for: Reduced Oligodendrocyte Density and Axonal Caliber Associated With Mitochondrial Alterations in the White Matter of Chronically‐Starved Mice
Source: Int J Eat Disord. 2026 Jan 16;59(5):869–84. doi: 10.1002/eat.70036 (PMC13147147; doi:10.1002/eat.70036)
Supplement: Supplementary file 1 — Data S1: Supporting Information. [file EAT-59-869-s001.pdf]

Supplementary Data S1

2. Method

2.2. Study Design

To evaluate running wheel activity (RWA), running wheels with a diameter of 11.5 cm were attached to the top of the cages as previously described (Gabloffsky et al., 2022; Staffeld et al., 2023). The covered distance per hour per individual mouse was estimated using the VitalView Activity software (VitalView Activity 1.4, STARR Life Science Corp.). Hyperactivity was defined as a significant increase in running activity, indicated by RWA within 24 hours during the different starvation phases.

For analysis of estrous cycle, staining of vaginal smears with 10% (v/v) Giemsa solution (Giemsa stock solution, ROTH T862.1, Karlsruhe, Germany) was conducted. Via microscopical analysis the vaginal smears were assigned to the different phases of the estrous cycle: estrous (fertile), metestrous, diestrous and proestrous. If there was no fertile phase within four days (the regular duration of the estrous cycle of mice), the cycle was classified as amenorrhea.

At the beginning of the acclimatization phase, the age of the mice corresponds to 28 days postnatal (P28), the end of the acclimatization phase corresponds to P38, the end of the acute starvation to P44, and the end of the chronic starvation phase to P57.

2.3. Immunohistochemistry and Image Analysis

Epitope retrieval was performed by heat-induced antigen retrieval in Tris/EDTA buffer (pH 9.0). Subsequently, the sections were incubated in 5% normal goat serum (NGS) in PBS for 1 h to block unspecific binding of the antibodies. The sections were incubated in the primary antibody diluted in NGS in a wet chamber at 4°C overnight. The next day, after washing with phosphate-buffered saline, the incubation with secondary antibody diluted in NGS was performed in a wet chamber for 1 h. For visualization of the antibody-labelling, the sections were incubated with 3,3-diaminobenzidine (Agilent DAKO, Santa Clara, CA, USA) for 10 min. To finish the staining, the sections were dehydrated and embedded with Depex.

For staining intensity measurements, the same slides were used as for the cell density measurements. The pixel classifier of QuPath was trained to detect the stained area above a specific threshold and measure the staining intensity in % per total area of the Region of interest. For training, 7 randomly selected segments of all scans were used, and the quality was evaluated subjectively for every analyzed Region of interest.

2.5. Ultrastructural Analysis

After dissection, the brains were embedded in 2.5% agarose. Coronal brain sections of 300 µm thickness were prepared using a vibratome (Leica VT1000 S, Leica, Wetzlar, Germany). After washing in 0.1 M phosphate-buffered saline, the sections were post-fixed for 2 hours with a 1% osmium tetroxide solution (OsO_4, Carl Roth, Germany), washed in distilled water, dehydrated in an ascending series of acetone, and infiltrated with Epon resin (Serva, Heidelberg, Germany), starting with a 1:1 mixture of acetone and resin overnight. Following infiltration in pure resin, the specific target areas were cut from the processed vibratome sections, flat-embedded in rubber molds filled with resin, and cured in an oven at 60°C for two days. Furthermore, surfaces of embedded tissues were exposed from the resin block with an ultramicrotome (Leica EM UC7, Leica, Wetzlar, Germany) using a diamond knife (Diatome, Nidau, Switzerland) for sectioning. Histology on toluidine blue-stained semithin sections (0.5-1.0 µm) enabled the selection of the Cingulum. Ultrathin sections (50-70 nm) were transferred to 300-mesh copper grids and contrasted with uranyl acetate and lead citrate for TEM. TEM imaging was performed using a Zeiss EM902 (Carl Zeiss Microscopy, Jena, Deutschland) operated at 80 kV equipped with a side-mounted 1x2k FT-CCD Camera (Proscan, Scheuring, Germany) using iTEM camera control and imaging software (iTEM version number 1187, Olympus Soft Imaging Solutions, Münster, Germany). Additionally, for the quantification of larger tissue areas, i.e., whole grid meshes, a field emission scanning electron microscope (Zeiss Merlin VP compact, Carl Zeiss Oberkochen, Germany) was used. Here, the scanning transmission electron microscopy (STEM) mode with combined dark-field and bright-field detection at an acceleration voltage of 15kV and a working distance of approximately 2.7mm was used to acquire high-resolution images (3072x2304 and 4096x3072 pixels, respectively) on carbon coated grids (3 nm, CCU-010-HV, Safematic, Zizers, Switzerland) covering an area of approximately 4000 µm² each.

To estimate expected mitochondria density by Disector's Principle (Cruz-Orive, 1987), a correction factor (Cf) was calculated first using the mitochondrial area of SIH_Morphology (A_{SIH}) and Control_Morphology ($A_{Control}$) (1). Afterwards the mitochondria density of Control_Morphology ($D_{control}$) was multiplied with Cf to receive the expected mitochondria density (D_{Exp}) (2). To estimate the corrected increase (I) the quotient of the observed mitochondria density in SIH_Morphology (D_{SIH}) and D_{Exp} was calculated and subtracted with 1 (3).

$$Cf = \frac{A_{SIH}}{A_{Control}} \qquad (1)$$

$$D_{exp} = D_{control} \times Cf \qquad (2)$$

$$I = \frac{D_{SIH}}{D_{Exp}} - 1 \qquad (3)$$

2.6. Statistics

For the mice perfused after chronic starvation (Control_Chronic_IHC and SIH_Chronic_IHC) and after refeeding (Control_Refeeding_IHC and SIH_Refeeding_IHC) for immunohistochemistry, the sample size calculation was based on serum neurofilament light chain (NfL) concentration as target variable (Zimmermann et al., 2025). Serum NfL concentration of SIH animals was increased compared to control animals (SIH: 72 pg/ml, SD: 28.56; Control: 28.49; SD: 15.89). This resulted in an effect size of Cohen's d: 1.88 and Cohen's f: 1.229. Using the G-Power software, a total of four animals per group were required (Lenhard & Lenhard, 2017). Two (Control_Chronic_IHC and SIH_Chronic_IHC), respectively one (Control_Refeeding_IHC and SIH_Refeeding_IHC) additional animals were included to account for potential dropouts. Thus, in total six (Control_Chronic_IHC and SIH_Chronic_IHC) and five (Control_Refeeding_SIH and SIH_Refeeding_IHC) were used.

For the mice perfused after chronic starvation for ELISA analysis (Control_Chronic_ELISA and SIH_Chronic_ELISA), the sample size calculation was based on running wheel activity as target variable (Frintrop et al., 2019). Running wheel activity of SIH animals increased significantly during acute starvation compared to the control group (SIH: 430.91%, SD: 260.44; Control: 234.65%, SD: 144.55). This resulted in an effect size of Cohen's d: 0.944 and Cohen's f: 0.472. Using G-Power software, a total of eight animals per group were required (Lenhard & Lenhard, 2017). Two additional animals were included to account for potential dropouts. Thus, in total ten animals were used.

For the mice perfused after chronic starvation for TEM analysis (Control_Morphology and SIH_Morphology), the sample size calculation was based on our previous study (Zimmermann et al., 2025) and is provided in the supplementary material of that publication (Zimmermann et al., 2025). For this cohort, the sample size calculation was based on brain atrophy as target variable (Frintrop et al., 2019). Brain volume of SIH animals was reduced by 9% compared to the control group (SIH: 188.98 mm^3, SD: 5.07; Control: 206.51 mm^3, SD: 3.61). This resulted in an effect size of Cohen's d: 1.24 and Cohen's f: 0.62. Using the G-Power software, we calculated that nine animals per group were required (Lenhard & Lenhard, 2017). Two additional animals were included to account for potential dropouts, resulting in a total of eleven animals.

For the mice used for ELISA analysis after refeeding (Control_Refeeding_ELISA and SIH_Refeeding_ELISA), the sample size calculation was based on brain atrophy defined as target variable (Frintrop et al., 2019). Brain volume of SIH animals was reduced by 9% compared to the control group (SIH: 188.98 mm^3, SD: 5.07; Control: 206.51 mm^3, SD: 3.61). This resulted in an effect size of Cohen's d: 1.24 and Cohen's f: 0.62. Using G-Power software, a total of nine animals per group were required (Lenhard & Lenhard, 2017). Two additional animals were included to account for potential dropouts, resulting in a total of eleven animals.

3. Results

3.1. Chronic starvation leads to the AN-related symptoms of hyperactivity and amenorrhea

SIH mice used for TEM analysis reached a 25% body weight loss by day 15. During the acute starvation phase, these SIH mice showed a trend toward increased running-wheel activity, and during the chronic starvation phase they exhibited a significant increase in running activity. Further, none of the SIH mice used for TEM analysis had a regular estrous cycle from the 5th block on throughout the whole chronic starvation phase (Supplementary Data Table S1).

The chronic starvation group for immunohistochemistry and the refeeding group for insulin-like growth factor 1 (IGF-1) investigation were previously described in (Zimmermann et al., 2025), while the chronic starvation group for IGF-1 evaluation was previously described in (Staffeld et al., 2023).

Table S1: Biometric Data of mice used for TEM analysis.

**Body weight**						
**Phase**	**Group**	**N**	**Mean [g]**	**SD**	**p-value**	**Cohen's d**
Acclimatization	Control	3	15.11	0.22	p = 0.09	1.844
	SIH	3	16.28	0.87		
Acute starvation	Control	3	15.64	0.20	$p \leq 0.01$	-4.469
	SIH	3	13.37	0.69		
Chronic starvation	Control	3	17.39	0.42	$p \leq 0.001$	-8.626
	SIH	3	12.04	0.77		
**Running activity**						
**Phase**	**Group**	**N**	**Mean [km]**	**SD**	**p-value**	**Cohen's d**
Acclimatization	Control	3	3.23	1.01	p = 0.43	-0.712
	SIH	3	2.50	1.04		
Acute starvation	Control	3	3.07	0.34	p = 0.07	2.046
	SIH	3	6.72	2.5		
Chronic starvation	Control	3	4.06	1.11	$p \leq 0.05$	3.669
	SIH	3	8.06	1.07		
**Incidence of estrous cycle**						
**Phase**	**Group**	**N**	**Incidence [%]**	**χ^2**	**p-value**	
1	Control	3	100	2.55	p = 0.11	
	SIH	3	66.67			
2	Control	3	0	0	p = 1	
	SIH	3	0			
3	Control	3	100	0	p = 1	

	SIH	3	100		
4	Control	3	100	2.55	p = 0.11
	SIH	3	66.67		
5	Control	3	100	21	$p \leq 0.001$
	SIH	3	0		
6	Control	3	100	21	$p \leq 0.001$
	SIH	3	0		
7	Control	3	100	21	$p \leq 0.001$
	SIH	3	0		
8	Control	3	100	21	$p \leq 0.001$
	SIH	3	0		

References

Cruz-Orive, L. M. (1987). Particle number can be estimated using a disector of unknown thickness: The selector. *Journal of Microscopy*, *145*(Pt 2), 121–142. https://pubmed.ncbi.nlm.nih.gov/3553604/

Frintrop, L., Trinh, S., Liesbrock, J., Leunissen, C., Kempermann, J., Etdöger, S., Kas, M. J., Tolba, R., Heussen, N., Neulen, J., Konrad, K., Päfgen, V., Kiessling, F., Herpertz-Dahlmann, B., Beyer, C., & Seitz, J. (2019). The reduction of astrocytes and brain volume loss in anorexia nervosa-the impact of starvation and refeeding in a rodent model. *Translational Psychiatry*, *9*(1), 159. https://doi.org/10.1038/s41398-019-0493-7

Gabloffsky, T., Gill, S., Staffeld, A., Salomon, R., Power Guerra, N., Joost, S., Hawlitschka, A., Kipp, M., & Frintrop, L. (2022). Food Restriction in Mice Induces Food-Anticipatory Activity and Circadian-Rhythm-Related Activity Changes. *Nutrients*, *14*(24). https://doi.org/10.3390/nu14245252

Lenhard, W., & Lenhard, A. (2017). Computation of different effect sizes like d, f, r and transformation of different effect sizes: Psychometrica.

Staffeld, A., Gill, S., Zimmermann, A., Böge, N., Schuster, K., Lang, S., Kipp, M., Palme, R., & Frintrop, L. (2023). Establishment of a Murine Chronic Anorexia Nervosa Model. *Cells*, *12*(13). https://doi.org/10.3390/cells12131710

Zimmermann, A., Rupprecht, H., Lang, S., Wienecke, R., Henschke, H.-S., Dickert, K., Schuster, K., Staffeld, A., Berger, C., Dück, A., Kölch, M., Vogelgesang, A., Grothe, M., Heinig, L., Wenzel, L., Kipp, M., & Frintrop, L. (2025). Increased Serum Neurofilament Light Chain Concentration Associated With Microglial Morphology Changes in Chronically-Starved Mice. *International Journal of Eating Disorders.* Advance online publication. https://doi.org/10.1002/eat.24423
